# Epstein-Barr Virus Infection Promotes Epithelial Cell Growth by Attenuating Differentiation-Dependent Exit from the Cell Cycle

**DOI:** 10.1128/mBio.01332-19

**Published:** 2019-08-20

**Authors:** Mark R. Eichelberg, Rene Welch, J. Tod Guidry, Ahmed Ali, Makoto Ohashi, Kathleen R. Makielski, Kyle McChesney, Nicholas Van Sciver, Paul F. Lambert, Sündüz Keleș, Shannon C. Kenney, Rona S. Scott, Eric Johannsen

**Affiliations:** a Department of Medicine, Division of Infectious Diseases, University of Wisconsin, Madison, Wisconsin, USA; b Department of Oncology, McArdle Laboratory for Cancer Research, University of Wisconsin, Madison, Wisconsin, USA; c Department of Biostatistics and Medical Informatics, University of Wisconsin, Madison, Wisconsin, USA; d Department of Microbiology and Immunology, LSUHSC-S, Shreveport, Louisiana, USA; e Department of Statistics, University of Wisconsin, Madison, Wisconsin, USA; Princeton University

**Keywords:** EBV, NPC, differentiation, gastric cancer, oral keratinocytes

## Abstract

Epstein-Barr virus (EBV) is a human herpesvirus that is associated with lymphomas as well as nasopharyngeal and gastric carcinomas. Although carcinomas account for almost 90% of EBV-associated cancers, progress in examining EBV’s role in their pathogenesis has been limited by difficulty in establishing latent infection in nontransformed epithelial cells. Recently, EBV infection of human telomerase reverse transcriptase (hTERT)-immortalized normal oral keratinocytes (NOKs) has emerged as a model that recapitulates aspects of EBV infection *in vivo*, such as differentiation-associated viral replication. Using uninfected NOKs and NOKs infected with the Akata strain of EBV (NOKs-Akata), we examined changes in gene expression due to EBV infection and differentiation. Latent EBV infection produced very few significant gene expression changes in undifferentiated NOKs but significantly reduced the extent of differentiation-induced gene expression changes. Gene set enrichment analysis revealed that differentiation-induced downregulation of the cell cycle and metabolism pathways was markedly attenuated in NOKs-Akata relative to that in uninfected NOKs. We also observed that pathways induced by differentiation were less upregulated in NOKs-Akata. We observed decreased differentiation markers and increased suprabasal MCM7 expression in NOKs-Akata versus NOKs when both were grown in raft cultures, consistent with our transcriptome sequencing (RNA-seq) results. These effects were also observed in NOKs infected with a replication-defective EBV mutant (AkataΔRZ), implicating mechanisms other than lytic-gene-induced host shutoff. Our results help to define the mechanisms by which EBV infection alters keratinocyte differentiation and provide a basis for understanding the role of EBV in epithelial cancers.

## Abstract (precis)

Latent infection by Epstein-Barr virus (EBV) is an early event in the development of EBV-associated carcinomas. In oral epithelial tissues, EBV establishes a lytic infection of differentiated epithelial cells to facilitate the spread of the virus to new hosts. Because of limitations in existing model systems, the effects of latent EBV infection on undifferentiated and differentiating epithelial cells are poorly understood. Here, we characterize latent infection of an hTERT-immortalized oral epithelial cell line (NOKs). We find that although EBV expresses a latency pattern similar to that seen in EBV-associated carcinomas, infection of undifferentiated NOKs results in differential expression of a small number of host genes. In differentiating NOKs, however, EBV has a more substantial effect, reducing the extent of differentiation and delaying the exit from the cell cycle. This effect may synergize with preexisting cellular abnormalities to prevent exit from the cell cycle, representing a critical step in the development of cancer.

## INTRODUCTION

Epstein-Barr virus (EBV) is a herpesvirus that infects over 90% of the population by adulthood. Primary EBV infection usually presents as a nonspecific illness in early childhood but often manifests as infectious mononucleosis in adolescents. Thereafter, EBV establishes lifelong latent infection in B lymphocytes. Rarely, EBV latent infection results in malignancy, including Burkitt and Hodgkin lymphoma, lymphoproliferative disease, nasopharyngeal carcinoma, and gastric cancer. Much of our knowledge of the transforming effects of latent EBV genes derives from the study of latent EBV infection of B lymphocytes *in vitro*, which results in their growth transformation into lymphoblastoid cell lines (LCLs). Extensive investigation of LCLs has demonstrated that latent genes constitutively activate growth and survival signals essential for normal B cell development by inducing CD40-like and B cell receptor-like signaling (reviewed in reference 1).

To elucidate the mechanisms by which EBV promotes the transformation of normal epithelial cells into carcinomas, it is important to study latent EBV infection in nontransformed cells. While LCLs have been useful models for investigating EBV lymphomas, there is no analogous epithelial cell transformation model. Since EBV infection of primary epithelial cells typically results in lytic infection (2, 3), the study of latent epithelial infection has been largely limited to infection of cultured carcinoma cell lines. These cell lines are of limited utility in understanding the role of latent EBV in the development of carcinomas because they cannot model critical premalignant virus-host interactions. Recently, the normal oral keratinocyte (NOK) model has emerged as a nontransformed epithelial cell that is capable of supporting latent EBV infection (4).

The NOK cell line was derived by expressing human telomerase reverse transcriptase (hTERT) in cells derived from gingival tissues obtained from an oral surgery (5). These cells display a normal morphology, have a functional p53 pathway, and retain the ability to differentiate. When NOKs infected with the Akata EBV strain (NOKs-Akata) are grown in organotypic raft cultures, EBV replication is observed in the differentiated layers (6). This propensity to replicate in more-differentiated epithelial cells has been observed in clinical specimens as well as in experimentally infected primary keratinocytes (3, 7). The ability of NOKs-Akata to support differentiation-dependent EBV replication has been crucial to the identification of cellular transcription factors that link these processes. For example, expression of KLF4 and BLIMP1, which promote epithelial differentiation, has been shown to induce expression of the EBV BRLF1 (Rta) and BZLF1 (Zta) immediate early genes, initiating the lytic cascade (6, 8). Recently, we have shown that EBV LMP1 is directly upregulated by cellular differentiation, independently of Rta and Zta, and in fact appears to enhance Rta and Zta differentiation-dependent expression (9).

The NOKs-Akata system has also revealed effects that EBV exerts on host cells. Birdwell et al. characterized genome-wide changes in DNA methylation associated with transient EBV infection (10). Cells that had been infected displayed stable hypermethylation of CpG islands, a common characteristic in EBV-positive carcinomas. EBV infection has also been observed to alter the morphology of NOKs grown in raft cultures, resulting in disruption of the suprabasal layers and decreased expression of some makers of differentiation, such as K10 (6). In addition, in EBV-infected cells, BrdU incorporation is no longer restricted to the basal layer of cells, although it is unclear if this is due to cellular DNA replication, viral DNA replication, or both (11). Finally, EBV infection of NOKs has been associated with disruption of the basal layer, with “invasion” of some keratinocytes into the collagen matrix. This phenomenon has been linked to LEF1 upregulation (12). The EBV factors responsible for these effects, including the extent to which they are attributable to latent versus lytic EBV genes, are unknown.

In the present study, we report an analysis of global gene expression changes caused by EBV infection in NOKs-Akata. By using this unbiased approach, we expected to identify perturbations of cellular signaling pathways induced by EBV infection and to associate those changes with phenotypes previously associated with EBV infection that potentially contribute to carcinomagenesis. Because EBV gene expression is linked to differentiation, we analyzed both EBV and host cell gene expression in undifferentiated NOKs and upon differentiation by methylcellulose. Finally, using a replication-defective EBV (AkataΔRZ), we were able to distinguish the effects of EBV latent infection from those induced by viral replication.

## RESULTS

### EBV gene expression in NOKs-Akata resembles that seen in EBV-positive carcinomas.

**FIG 1:**
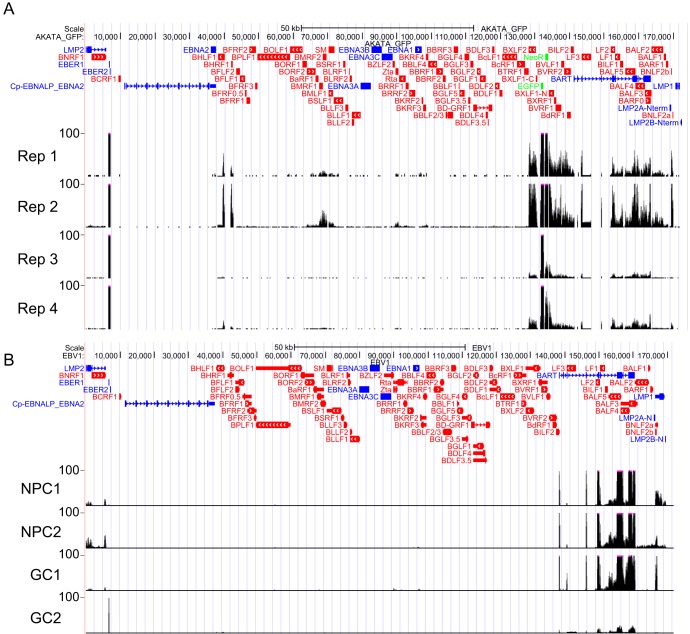
EBV RNA expression in NOKs-Akata reflects that observed in EBV-positive carcinomas. (A) RNA-seq reads from undifferentiated NOKs-Akata mapping to the EBV genome are shown for each replicate (Rep). Track height represents RPKM. Genes corresponding to observed signals are indicated below the tracks. (B) RNA-seq reads from published NPC and EBV-positive gastric tumor samples mapping to the EBV genome are shown. Track height represents read depth.

To examine the global changes in gene expression induced by EBV infection and differentiation, we analyzed NOKs and NOKs-Akata by transcriptome sequencing (RNA-seq). Each cell line was either grown as an undifferentiated monolayer or differentiated by suspension in methylcellulose (MC) for 24 h. Four replicates were performed for each condition from independent seedings of cells. Reads were aligned to the human genome (hg19) and to the Akata EBV strain as detailed in Materials and Methods. EBV gene expression in NOKs-Akata included the noncoding EBV-encoded RNAs (EBERs) and BamHI-A rightward transcripts (BARTs) (Fig. 1A). A variable degree of spontaneous lytic gene expression was observed in each replicate (with the strongest signals at the OriLyt, BMRF1/BMLF1, and BNLF2a/BNLF2b loci). In the absence of a differentiation stimulus, we detected very few reads mapping to the LMP1 and LMP2A/B transcripts, other than reads attributable to BNLF2a/BNLF2b, which overlap the 3′ untranslated region (3′ UTR) of the LMP1 transcript.

To compare EBV gene expression in NOKs-Akata to that observed in EBV-associated carcinomas, we aligned reads to the EBV genome from publicly available RNA-seq data sets of four nasopharyngeal carcinoma (NPC) (13) and 27 EBV-associated gastric cancer (EBVaGC) (14) tumors. As expected, high levels of the BART RNAs were observed. We did not reliably detect EBER expression in these samples, likely because these RNA-seq libraries were prepared from poly(A)-enriched RNA, whereas our libraries used ribodepletion. As with NOKs-Akata, there were varied amounts of expression of lytic genes. Latent membrane protein expression was highly varied; in general, LMP1 was not expressed or was expressed at very low levels in GC tumors but was present in 3 out of 4 NPC tumors (Fig. 1B; see also Fig. S1 in the supplemental material). LMP2A/B was expressed in 16 out of 27 GC tumors and in all four NPC tumors. In addition, the BNLF2a/BNLF2b transcript was detected in 13 of 27 GC and 3 of 4 NPC tumors, which includes tumors with little or no spontaneous lytic replication. In summary, latent EBV gene expression levels varied in the tumor samples that we analyzed but conformed to a latency I/II pattern and closely resembled that observed in our NOKs-Akata model.

10.1128/mBio.01332-19.4 FIG S1 EBV gene expression in EBV-positive carcinomas. Normalized read depths are shown for 4 EBV-positive nasopharyngeal carcinoma (NPC) tumors and 27 EBV-associated gastric cancer (GC) tracks. Although each track is presented at the same scale, differences in apparent expression level may also be attributable to the presence of nontumor cells (e.g., stromal or tumor infiltrating lymphocytes) in the sequenced samples. Download FIG S1, EPS file, 2.7 MB. Copyright © 2019 Eichelberg et al. 2019 Eichelberg et al. This content is distributed under the terms of the Creative Commons Attribution 4.0 International license.

### Differentiation affects the expression of EBV genes.

**FIG 2:**
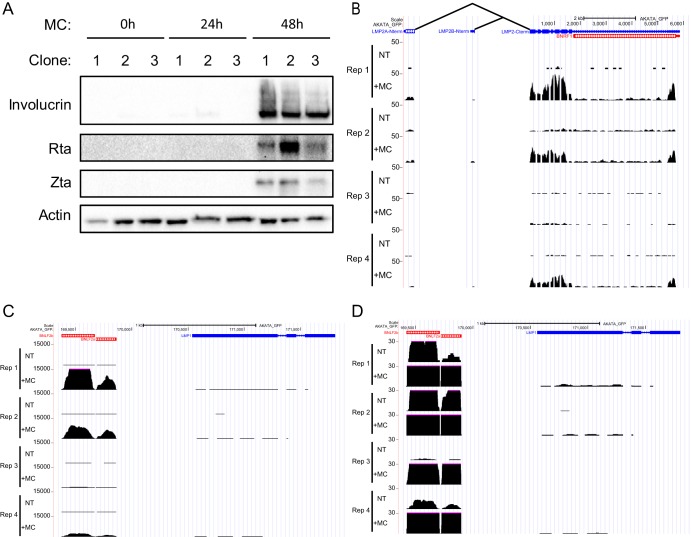
Effect of differentiation on EBV gene expression in NOKs-Akata. (A) Western blots for the indicated EBV and cell proteins from undifferentiated NOKs-Akata or upon differentiation with methylcellulose (MC) are shown. RNA-seq reads mapping to the Akata genome from undifferentiated NOKs-Akata (NT) and upon differentiation with MC are shown for the following genes: *LMP2A* (B), *BNLF2a* and *BNLF2b* (C), and *LMP1* (D). Track height represents RPKM.

We and others have previously shown that differentiation of NOKs-Akata by a variety of stimuli promotes entry into the lytic cycle (6, 11), and we again observed this in our MC-treated NOKs-Akata (Fig. 2A). Induction into the lytic cycle was more apparent after 48 h than at the 24-h time point, when RNA-seq was performed. Our RNA-seq results were remarkable for strong induction of LMP2A/B upon NOK differentiation (Fig. 2B) in 3 of 4 replicates. Examination of the unique 5′ exons suggests that this was predominantly the LMP2A transcript, though lower levels of LMP2B exon 1 were present in these three replicates. The BNLF2a/BNLF2b transcript was upregulated by several orders of magnitude upon differentiation (Fig. 2C and D). In contrast, under these conditions, we observed minimal induction of the LMP1 transcript upon differentiation of NOKs-Akata (Fig. 2D).

### Few host genes are differentially regulated in NOKs infected with EBV.

**FIG 3:**
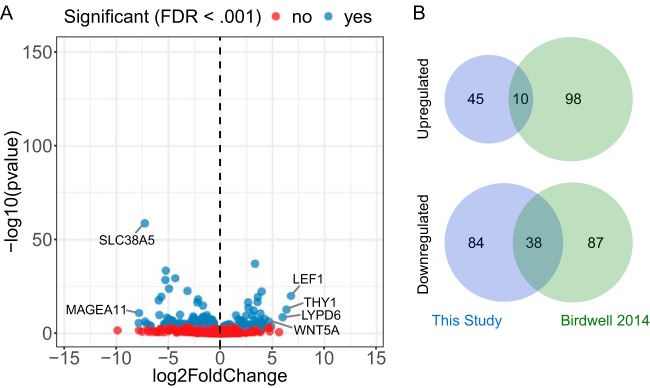
Few genes are differentially expressed between NOKs and NOKs-Akata. (A) Volcano plot showing genes upregulated or downregulated by EBV infection in NOKs-Akata relative to those in uninfected NOKs. Differentially expressed genes (false-discovery rate [FDR] < 0.001) are indicated with blue dots, whereas nondifferentially expressed genes are shown in red. (B) Venn diagrams comparing genes observed to be regulated by EBV infection in our RNA-seq data compared with those previously reported to be regulated by transient EBV infection.

To determine host cell changes induced by EBV infection of undifferentiated NOKs, we compared normalized expression levels for all detected transcripts using DESeq2 (Fig. 3A; Table S2). This identified 55 genes upregulated and 122 genes downregulated by EBV infection (≥1.8-fold change, *P* < 0.05). Although these observed changes were small in magnitude and few in number, we observed the overlap of several genes previously identified as regulated by transient EBV infection in the same model (Fig. 3B) (10). The overlap was more extensive for downregulated genes (38 of 122), whereas only 10 of 55 upregulated genes were common between the two data sets.

10.1128/mBio.01332-19.2 TABLE S2 Genes differentially expressed in NOKs and NOKs-Akata. All genes identified by RNA-seq as differentially expressed (fold change > 1.8, *P* < 0.05) between undifferentiated NOKs-Akata and NOKs are listed, along with the ratio of expression, calculated by dividing the number of transcripts per million for NOKs-Akata (TPM_NOKs-Akata_) by that for TPM_NOKs_. Genes previously reported to be regulated by transient EBV infection are also listed, and genes common to both sets are identified. Download Table S2, XLS file, 0.04 MB. Copyright © 2019 Eichelberg et al. 2019 Eichelberg et al. This content is distributed under the terms of the Creative Commons Attribution 4.0 International license.

**FIG 4:**
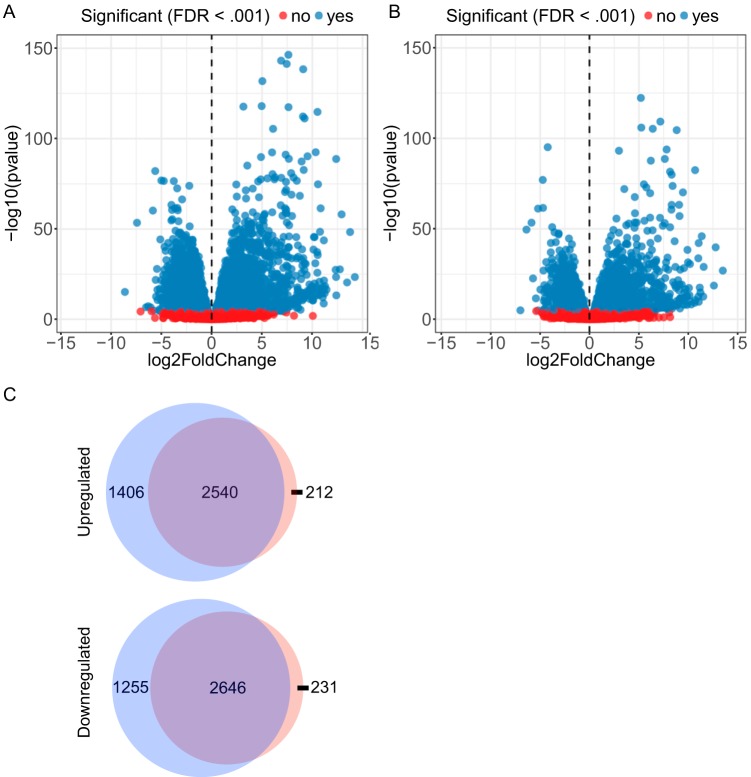
Differentiation causes substantially larger effects on gene expression than EBV infection of undifferentiated NOKs. Volcano plots show changes in gene expression induced by differentiation with MC in NOKs (A) or NOKs-Akata (B). Genes that are significantly changed (FDR < 0.001) are represented by blue dots. (C) Venn diagrams of the numbers of genes differentially expressed with MC (adjusted *P* < 0.01) in NOKs (blue circles) and NOKs-Akata (red circles).

We also examined host gene expression induced by differentiation with MC for 24 h. Examination of several genes known to be activated during keratinocyte differentiation revealed strong induction of early markers, such as involucrin and cornifins (Table 1). However, the late differentiation markers loricrin and filaggrin were not expressed, probably a reflection of the 24-h time point chosen for our analysis. Interestingly, across all early-differentiation markers measured, expression was more strongly induced in NOKs than in NOKs-Akata. Unlike with EBV infection, differentiation resulted in differential expression of thousands of cell genes (Fig. 4A and B). In NOKs, there were 3,946 upregulated and 3,901 downregulated genes with MC treatment. In NOKs-Akata, somewhat smaller numbers of genes were differentially regulated, with 2,752 upregulated and 2,877 downregulated genes. The majority of genes affected by differentiation are common to both NOKs and NOKs-Akata (Fig. 4C). Although EBV had a limited effect on gene expression in undifferentiated NOKs-Akata, differences were much greater in the MC-differentiated state. Compared with differentiated NOKs, differentiated NOKs-Akata had 684 upregulated and 678 downregulated genes.

**TABLE 1:** RNA-seq quantification of genes associated with early and late keratinocyte differentiation

Differentiation stage	Gene name	Mean expression level (in TPM)^*a*^:			
		NOKs		NOKs-Akata	
		Undiff.	+MC	Undiff.	+MC
Late	Loricrin	0.0	0.1	0.0	0.1
	Filaggrin	0.2	0.5	0.3	1.2
Early	Involucrin	0	99	0	12
	Cornifin 1A	1	1,824	0	836
	Cornifin 1B	18	5,587	3	3,421
	Cornifin 2A	2	341	0	148
	Cornifin 2B	3	206	1	157
	Cornifin 3	0	2,040	0	280
	Transglutaminase 1	1	715	1	408
	Transglutaminase 3	0	41	0	8
	Transglutaminase 5	0	40	0	10

a TPM, number of transcripts per million. Values are means for undifferentiated (undiff.) or MC-differentiated (+MC) NOKs or NOKs-Akata for each gene, grouped according to association with early or late keratinocyte differentiation.

### Differentiation of both NOKs and NOKs-Akata leads to changes in the same signaling pathways, although these changes are attenuated in NOKs-Akata.

**FIG 5:**
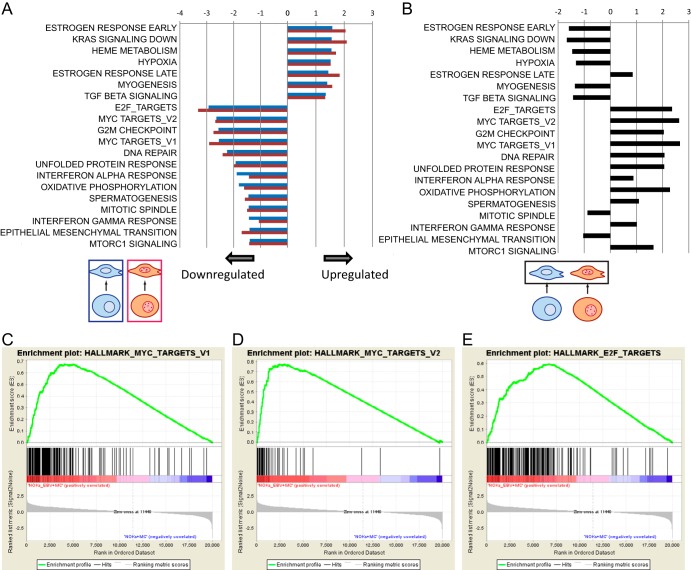
Pathway analysis identifies downregulation of cell cycle and proliferation signaling pathways after induction of differentiation in both NOKs and NOKs-Akata. (A) Gene set enrichment analysis (GSEA) was performed using the hallmark gene set collection from MSigDB. Normalized enrichment scores (NES) for pathways that were significantly different (*q* < 0.05) between undifferentiated NOKs and differentiated NOKs are displayed with blue bars. NES for the changes in the same pathways comparing undifferentiated NOKs-Akata with differentiated NOKs-Akata are shown in red. Positive enrichment scores represent pathways that are activated by MC differentiation, and negative enrichment scores represent pathways that are suppressed by differentiation. (B) GSEA using hallmark gene sets found to be regulated by differentiation was used to compare differentiated NOKs against differentiated NOKs-Akata. NES for each pathway are shown with black bars. (C to E) GSEA enrichment plots of NOKs-Akata with MC versus NOKs with MC for Myc targets V1, Myc targets V2, and E2F targets gene sets. Genes are sorted by signal-to-noise ratios, with genes expressed more highly in NOKs-Akata treated with MC on the left.

To identify signaling pathways affected by MC treatment, we used gene set enrichment analysis (GSEA) with the MSigDB hallmark gene sets to characterize the regulation of genes in NOKs treated with MC and compared it with that of untreated NOKs. We observed strong downregulation of pathways associated with cell cycle progression or cell metabolism, including Myc signaling, E2F signaling, mammalian target of rapamycin complex 1 (mTORC1), and oxidative phosphorylation (Fig. 5A, blue bars). Although the MSigDB hallmark sets do not include a pathway specific to epithelial differentiation, we did observe upregulation of genes associated with transforming growth factor β (TGF-β) signaling, which has been reported to be essential for keratinocyte differentiation (15). The most strongly induced gene set was hallmark estrogen signaling early. Estrogen signaling is dependent on cell context and may contribute to both cell growth and differentiation (16–18). Closer inspection of the hallmark estrogen signaling early pathway revealed that several of the genes driving this signal are involved in epithelial cell differentiation, including *KLF4*, *HES1*, *SLC9A3R1*, *ELF3*, *BCL11B*, *KAZN*, *GJA1*, and *MREG*. On the other hand, several genes in this gene set that are associated with cell growth and survival were in fact downregulated by MC, including *CCND1*, *AREG*, *XBP1*, *SCARB1*, *IGFBP4*, *BCL2*, *RARA*, *FKBP4*, *MYC*, and *INPP5F1* (Fig. S2). Collectively, these data suggest that the estrogen signaling early signal results from genes associated with estrogen-induced differentiation rather than growth.

10.1128/mBio.01332-19.5 FIG S2 Identification of estrogen signaling-related genes associated with differentiation or cell growth. GSEA enrichment plot for the hallmark estrogen signaling early pathway. Genes upregulated by MC and overlapping Gene Ontology (GO) pathways associated with epithelial differentiation are listed on the left. Genes downregulated by MC and overlapping GO pathways associated with cell growth or cell survival are listed on the right. Listed genes fall within the indicated range. Genes are sorted by signal-to-noise ratio. Download FIG S2, EPS file, 1.5 MB. Copyright © 2019 Eichelberg et al. 2019 Eichelberg et al. This content is distributed under the terms of the Creative Commons Attribution 4.0 International license.

In raft cultures, EBV infection of NOKs results in irregular morphology and promotes invasion into the dermal equivalent (10–12). To examine how EBV may affect differentiation-induced signaling pathways, we repeated our GSEA of MC treatment with NOKs-Akata (Fig. 5A, red bars) and found a strong concordance with gene sets affected by MC in NOKs. This suggests that MC treatment affects the same signaling pathways irrespective of EBV, even though fewer genes were differentially expressed in NOKs-Akata. To further investigate how EBV modulates differentiation, we analyzed the genes differentially expressed between NOKs and NOKs-Akata after MC treatment. Remarkably, for almost all pathways affected by MC treatment, the effect of EBV infection was the opposite of the MC effect (Fig. 5B; Fig. S3 and S4). The three hallmark gene sets most strongly affected by EBV were Myc targets V1, Myc targets V2, and E2F targets, all of which are strongly downregulated by differentiation but remained elevated in NOKs-Akata relative to in NOKs (Fig. 5C to E).

10.1128/mBio.01332-19.6 FIG S3 Hallmark gene sets identified as upregulated in NOKs-Akata with MC versus NOKs with MC. GSEA enrichment plots are displayed for all hallmark gene sets identified as upregulated in NOKs-Akata plus MC compared with NOKs plus MC (*q* < 0.05). Download FIG S3, TIF file, 0.8 MB. Copyright © 2019 Eichelberg et al. 2019 Eichelberg et al. This content is distributed under the terms of the Creative Commons Attribution 4.0 International license.

**FIG 6:**
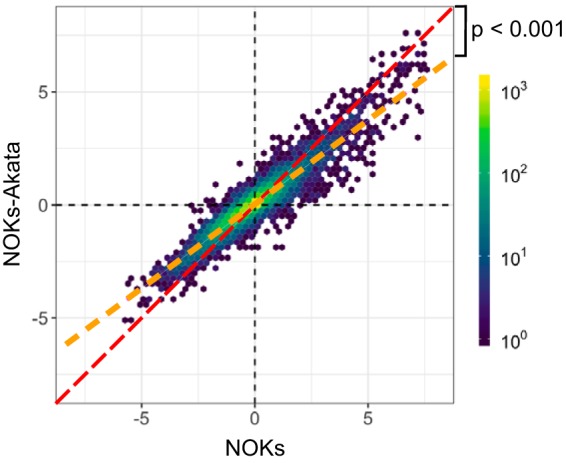
EBV infection impairs differentiation-induced changes in NOKs-Akata via a transcriptome-wide decrease in the magnitude of gene expression changes induced by differentiation. Hex bin plot displaying the response of gene expression to MC differentiation. All measured genes are plotted according to their log_2_ fold change in response to MC treatment in NOKs (*x* axis) and NOKs-Akata (*y* axis). Color represents the number of genes in each bin. The diagonal dashed line has a slope of 1, representing the expected result if differentiation produced identical gene changes in NOKs and NOKs-Akata.

Because differentiation is essential for EBV to complete its replication cycle, we speculated that EBV infection would alter a subset of differentiation-induced gene changes. In an effort to identify these genes, we compared for each gene the magnitude of the MC-induced change in NOKs with that of the MC-induced change in NOKs-Akata (Fig. 6). Across all genes measured, those induced by differentiation were more highly upregulated in NOKs than in NOKs-Akata, and likewise, genes downregulated by differentiation were more highly repressed in NOKs than in NOKs-Akata. In fact, we observed a nearly perfect correlation between the MC-induced gene changes (Fig. 6, orange dashed line), with very few genes deviating substantially from the diagonal. In order to identify genes that deviate significantly from this trend, we calculated the residuals from this linear model (see the histogram in Fig. S5). Using a likelihood ratio test, we found that 18 genes (Table S3) deviated significantly (*P* < 0.01). Notable among these was cyclin D3, whose upregulation in NOKs-Akata may account for global cell cycle effects of EBV upon differentiation.

10.1128/mBio.01332-19.3 TABLE S3 Genes deviating significantly from the linear model. In Fig. 6, expression levels of most genes were observed to change less in response to MC in NOKs-Akata than was observed in NOKs. To identify genes deviating significantly from this trend, we performed a likelihood ratio test with DESeq2 to compare the following models: a reduced model in which gene expression was approximately the infection state with treatment, and a full model in which gene expression was approximately the infection state with treatment and interaction (infection state, treatment). The 18 genes with adjusted *P* values (Benjamini-Hochberg) of <0.01 are present, along with their log_2_ fold change in response to MC. Residuals from the linear model are shown in column D. Download Table S3, XLS file, 0.04 MB. Copyright © 2019 Eichelberg et al. 2019 Eichelberg et al. This content is distributed under the terms of the Creative Commons Attribution 4.0 International license.

10.1128/mBio.01332-19.7 FIG S4 Hallmark gene sets identified as downregulated in NOKs-Akata with MC versus NOKs with MC. GSEA enrichment plots are displayed for all hallmark gene sets identified as downregulated in NOKs-Akata plus MC compared with NOKs plus MC (*q* < 0.05). Download FIG S4, TIF file, 2.8 MB. Copyright © 2019 Eichelberg et al. 2019 Eichelberg et al. This content is distributed under the terms of the Creative Commons Attribution 4.0 International license.

10.1128/mBio.01332-19.8 FIG S5 Residual histogram of the linear model represented in Fig. 6. The linear model was calculated as the difference between the observed NOKs-Akata average gene fold change and the predicted NOKs-Akata gene fold change as a linear function of the NOKs gene fold change. The histogram shows the distribution of these residuals across all measured genes. Download FIG S5, EPS file, 0.5 MB. Copyright © 2019 Eichelberg et al. 2019 Eichelberg et al. This content is distributed under the terms of the Creative Commons Attribution 4.0 International license.

### AkataΔRZ is a virus mutant that is unable to undergo lytic reactivation.

**FIG 7:**
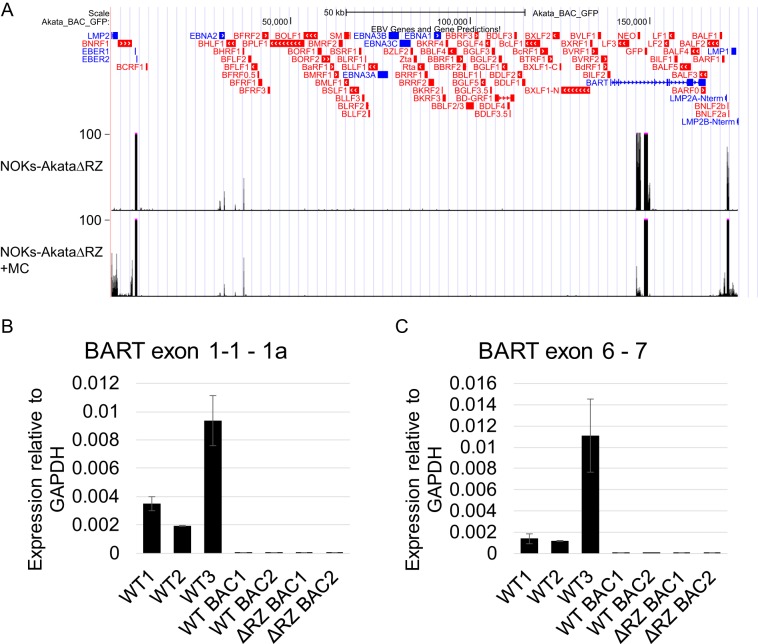
EBV gene expression from NOKs-AkataΔRZ. (A) RNA-seq reads mapping to the EBV genome from undifferentiated (NT) and MC-differentiated (MC) NOKs-AkataΔRZ. Genes corresponding to observed signals are indicated below the tracks. (B and C) Reverse transcription-qPCR assessing BART transcript expression from uninfected NOKs and NOKs infected with the indicated Akata mutant strains. GAPDH, glyceraldehyde-3-phosphate dehydrogenase; WT, wild type.

Because EBV lytic gene expression is activated by differentiation, we speculated that lytic gene effects, especially BGLF5-induced host shutoff (19), might be responsible for the blunting of differentiation-induced gene changes in infected cells. To determine the extent to which lytic gene expression contributes to the impaired differentiation seen in NOKs-Akata cells, we constructed a mutant virus with the BRLF1 and BZLF1 genes deleted (AkataΔRZ). These genes encode the immediate early proteins Rta and Zta, both of which are essential for EBV lytic cycle entry (20). As expected, AkataΔRZ was unable to express the lytic cascade in response to MC (Fig. 7A). Transfection of BRLF1 and BZLF1 restored the lytic cascade, including late gene expression (Fig. S6). Interestingly, the BNLF2a/BNLF2b genes were expressed as latent genes and were further upregulated by MC treatment. We observed MC-induced upregulation of LMP1, as we have previously reported, but also found that LMP2A was upregulated by MC-induced differentiation independently of Rta and Zta. Unexpectedly, the AkataΔRZ virus did not express detectable BART transcripts in NOK cells. To further investigate this observation, we established multiple independent NOK clones infected with the parental Akata bacmid and AkataΔRZ. We were unable to detect BART expression in any of these cells (Fig. 7B and C), suggesting that BART expression is defective in the original bacmid and not as a result of the BRLF1 and BZLF1 deletion. To account for this difference between our NOKs-Akata and NOKs containing a bacmid-derived virus, we used NOKs infected with the wild-type bacmid (NOKs-Akata-BAC) as a control in subsequent experiments.

10.1128/mBio.01332-19.9 FIG S6 Validation of the AkataΔRZ bacmid. (A) Deletion of BRLF1 and BZLF1 was verified using primers flanking the deletion (GTGTTGCAGTATGTACAGTTAGC and GTAATGGCATCCGTGACCTC). The gel demonstrates the expected PCR product size of 1,924 bp. (B) Western blot of the HeLa cells infected with the AkataΔRZ virus and transcomplemented with the BRLF1 and BZLF1 protein products Rta and Zta. Early (BMRF1) and late (VCAp18) gene products are readily detected at 72 h; the actin blot is shown as a loading control. Download FIG S6, EPS file, 2.0 MB. Copyright © 2019 Eichelberg et al. 2019 Eichelberg et al. This content is distributed under the terms of the Creative Commons Attribution 4.0 International license.

### Raft cultures of NOKs-Akata show impaired differentiation relative to that of NOKs even in the absence of EBV replication and BART expression.

**FIG 8:**
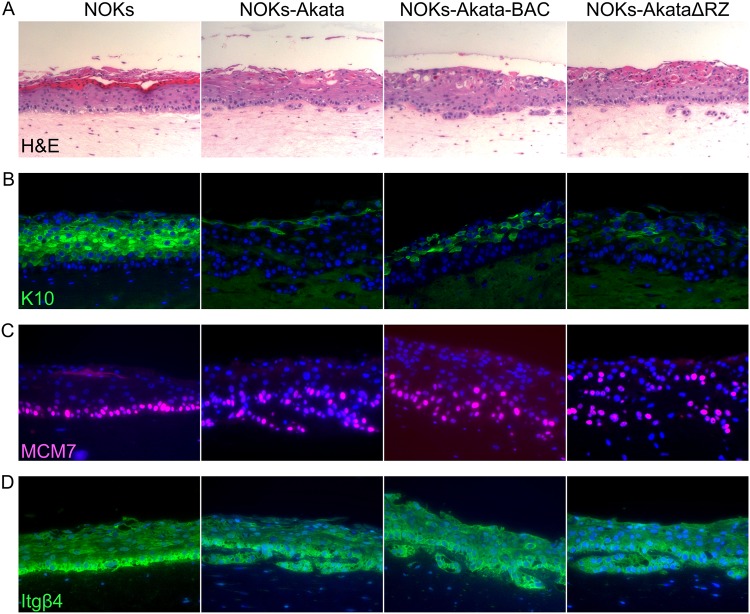
NOKs-Akata, NOKs-Akata-BAC, and NOKs-AkataΔRZ all exhibit an inhibited ability to differentiate. Three clones each of NOKs, NOKs-Akata, NOKs-Akata-BAC, and NOKs-AkataΔRZ were differentiated as raft cultures. Representative sections are shown for uninfected NOKs and NOKs infected with the indicated Akata mutant strains. Sections were stained with hematoxylin and eosin (H&E) (A), K10 (B), MCM7 (C), or ITGB4 and nuclei with Hoechst 33342 dye (D).

To assess which EBV gene products account for the attenuation of differentiation, we grew NOKs and NOKs infected with wild-type Akata, Akata-BAC, or AkataΔRZ in raft cultures. In the suprabasal layers of a raft culture, NOKs display strong expression of the differentiation-associated cytokeratin K10 (Fig. 8). NOKs-Akata have substantially reduced expression of K10 throughout the suprabasal layers, expressing it most strongly in cells along the apical edge. Both NOKs-Akata-BAC and NOKs-AkataΔRZ also show a reduced expression of K10 throughout the raft, similar to that observed with NOKs-Akata.

Because one of the key steps in keratinocyte differentiation is the end of cell cycle progression, we sought to investigate the proliferative potential of rafted cells by measuring the expression of the DNA replication licensing factor MCM7. This protein is highly expressed throughout the basal layer of NOKs but is rapidly lost in suprabasal keratinocytes. However, in NOKs-Akata, NOKs-Akata-BAC, and NOKs-AkataΔRZ, MCM7 remains expressed in a subset of suprabasal cells. In addition, some cells in rafts of all three virus-infected cell lines appear below the basal layer. This can be clearly observed when rafts are stained for integrin β4 (Itgβ4), which anchors basal keratinocytes to the basement membrane. While staining of Itgβ4 in NOKs indicates a single, relatively smooth layer of basal cells across the surface of the collagen raft, in the virus-infected rafts, it identifies invasive growth into the collagen matrix. The invasive phenotype is observed in NOKs-Akata, NOKs-Akata-BAC, and NOKs-AkataΔRZ (Fig. 8). Thus, NOKs infected with EBV strains defective for replication and/or BART expression exhibit the same aberrant growth behavior and impaired differentiation in raft cultures as those infected with wild-type Akata.

**FIG 9:**
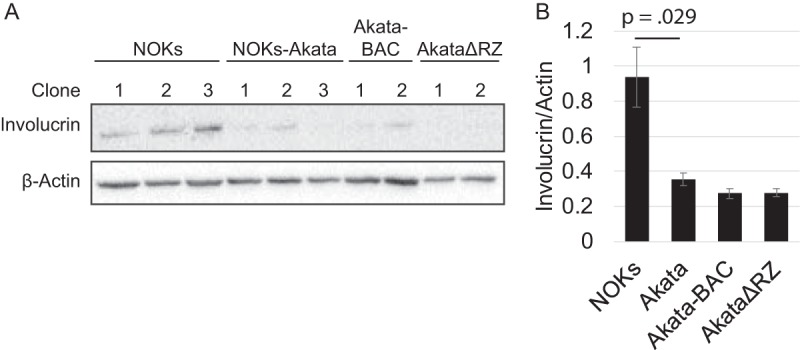
AkataΔRZ inhibits differentiation with MC. (A) Western blot showing involucrin expression in independently derived clones of NOKs, NOKs-Akata, NOKs-Akata-BAC, and NOKs-AkataΔRZ differentiated in MC for 24 h; (B) Quantification of the results shown in panel A as measured using ImageJ and normalized to β-actin. Bars indicate standard error.

To further assess the effects of these EBV mutations on differentiation, we assessed involucrin expression levels in three clones of NOKs, NOKs-Akata, NOKs-Akata-BAC, and two clones of NOKs-AkataΔRZ differentiated by MC for 24 h (Fig. 9). Although involucrin levels varied from clone to clone, we observed the highest levels in NOKs; levels were consistently lower in NOKs infected with any of our viruses (Akata, Akata-BAC, and AkataΔRZ).

## DISCUSSION

In this paper, we report the first global analysis of gene expression changes caused by EBV infection and differentiation in a nontransformed, immortalized keratinocyte model. In undifferentiated NOKs, we observed a pattern of EBV latent gene expression that mirrors that seen in EBV-associated carcinomas. The most prominently expressed genes are the EBER and BART genes, as well as BNLF2a/BNLF2b. When NOKs were differentiated by suspension in MC for 24 h, BNLF2a and BNLF2b expression increased. In addition, LMP2A expression was induced, and a minimal amount of *LMP1* expression was detectable. The dependence of these genes on cellular factors associated with differentiation likely explains their varied expression in EBV carcinomas. Specifically, LMP1 and LMP2A expression was more likely to be detected in NPC than in GC, but the levels of expression of both transcripts varied among tumor samples. LMP2A was detected in all four NPC tumors but in only 59% of GCs, while LMP1 was expressed in 3 out of 4 NPCs and mostly absent in GCs. The BNLF2a/BNLF2b transcript(s) was detected in all four replicates using NOKs and strongly induced by differentiation. We also observed the expression of BNLF2a/BNLF2b in 3 out of 4 NPCs analyzed, as well as 48% of GC tumors. This has been previously reported for GC tumors, where their expression was not correlated with lytic gene expression, suggesting that these genes may be latent in these tumors (21). Our observation of BNLF2a/BNLF2b expression in AkataΔRZ-infected cells lends additional support to this possibility.

Analysis of cellular transcripts in undifferentiated NOKs suggested that latent EBV infection had only subtle effects on host gene transcription. There was overlap, particularly among downregulated genes, between genes identified in the current RNA-seq study and those detected in a previous microarray-based study wherein NOKs were transiently infected by EBV (10). This overlap is noteworthy, as these studies had significant methodological differences. In particular, the microarray study analyzed four clonal subpopulations, whereas the present study compares a population of cells with ongoing infection and against the parental clone. Since EBV-induced methylation was proposed to be the major mechanism by which transient EBV infection exerts these changes, it is interesting to note that the overlap was greatest among EBV-downregulated genes. These downregulated genes include the cadherins *PCDH10*, *CDH11*, and *PCDHB6*, which may contribute to the invasive phenotype, as well as *DUOX1*, which has been identified as playing a significant role in the regulation of differentiation-induced genes in keratinocytes (22). Among those genes upregulated by EBV in both studies are *WNT5A* and *LEF1*, which have been identified for their role in increasing cell motility and invasiveness (12).

In contrast with the minor effects of EBV infection, differentiation with MC induced much more substantial changes in gene expression. Genes associated with early keratinocyte differentiation, such as cornifins and transglutaminases, were activated, in some cases by several hundred fold. Two late-differentiation genes, filaggrin and loricrin, were not detected, possibly because treatment for 24 h was not sufficient to allow their activation. To analyze the genes affected by differentiation, we used GSEA to identify the hallmark gene sets activated or downregulated by differentiation. Gene sets that were downregulated include E2F targets, Myc targets, and a G2M checkpoint. The downregulation of these gene sets is consistent with previously established signaling events during keratinocyte differentiation (23–26). Among gene sets upregulated during differentiation are those associated with an estrogen response. Estrogen signaling can be associated with either cell growth or cell differentiation. Closer inspection of the hallmark estrogen response early gene set revealed that several of the genes in this gene set that were upregulated were associated with differentiation and overlapped the Gene Ontology (GO) epithelial cell differentiation gene set. In contrast, genes in the estrogen response early gene set that are associated with cell proliferation and survival were downregulated by MC treatment. Since NOKs do not express the estrogen receptor at the mRNA level, it is likely that the hallmark estrogen response early gene set is not enriched due to estrogen signaling but because the gene set contains several genes that are expressed during keratinocyte differentiation.

Although MC treatment of both NOKs and NOKs-Akata upregulated or downregulated broadly similar genes, corresponding to enrichment of the same gene sets, there were significant differences in gene expression between NOKs and NOKs-Akata treated with MC. GSEA of the genes expressed in MC-treated NOKs and NOKs-Akata revealed a pattern that was largely the inverse of the effect of differentiation. That is, gene sets upregulated by MC treatment were lower in MC-treated NOKs-Akata than in MC-treated NOKs, and gene sets that are downregulated by differentiation are higher in NOKs-Akata compared with NOKs. The gene sets most significantly affected include two Myc target gene sets and E2F targets. These genes are strongly downregulated by differentiation as cells cease proliferation. Continued expression of these genes in NOKs-Akata likely represents a delayed exit from the cell cycle. The inverse effect of EBV on differentiation-regulated pathways indicates that the presence of EBV dampens the responsiveness to the differentiation stimulus. This is reflected in the expression levels of selected keratinocyte differentiation-associated genes, which were activated to a significantly lesser degree in NOKs-Akata. By measuring the responses of all genes to MC treatment in NOKs and comparing them with those in NOKs-Akata, we observed a broad, transcriptome-wide blunting of the differentiation effect. This suggests that EBV inhibits the differentiation process as a whole rather than disrupts only one pathway or a few signaling pathways associated with differentiation.

Our data show that the inhibition of differentiation is the most significant phenotype resulting from EBV infection of NOKs. Since differentiation of NOKs-Akata can also cause lytic reactivation of the virus, we hypothesized that expression of lytic genes may be responsible for inhibiting differentiation. To test this hypothesis, we used a recombinant virus missing the BRLF1 and BZLF1 genes, which are essential for transcription of lytic genes. The AkataΔRZ virus also did not express the BART transcript, a defect that we determined was also present in the parental bacmid virus. This may be due to the antisense insertion of the green fluorescent protein (GFP) and G418 resistance genes into a BART intron, interfering with BART transcription, unlike the insertion into the BXLF1 gene in the nonbacmid Akata. We observed the differentiation of NOKs, NOKs-Akata, NOKs-Akata-BAC, and NOKs-AkataΔRZ using organotypic raft cultures, with focus on the morphology of the rafts, expression of an early-differentiation marker (K10), and expression of a DNA replication licensing factor (MCM7). Morphologically, NOKs-Akata had less organized basal and suprabasal layers than NOKs. Some cells in the NOKs-Akata raft were observed to have grown downward into the collagen raft. This invasive behavior has been described previously (6). Invasive cell growth was readily observed when cells were stained for Itgβ4, which is strongly expressed in basal NOKs. We observed a significant degree of invasiveness across all samples of NOKs-Akata, NOKs-Akata-BAC, and NOKs-AkataΔRZ. The early differentiation marker K10 is expressed in NOKs even a single cell layer above the basal layer, and it remains expressed robustly throughout the suprabasal layers. Rafts infected with Akata, Akata-BAC, or AkataΔRZ all had substantially reduced expression of K10, and its expression was delayed until cells were more than one layer above the basal layer. In addition, some amount of MCM7 could be observed above the basal layer in all EBV-infected rafts. Taken together, these results are consistent with a delayed or inhibited differentiation in all EBV-infected rafts, regardless of the ability to express lytic genes.

Because the AkataΔRZ virus inhibited differentiation in a manner similar to that of the wild-type bacmid and nonbacmid viruses, our data suggest that EBV inhibits differentiation through a mechanism that does not require the expression of lytic genes or the BART microRNAs (miRNAs). Candidates that might mediate the inhibition of differentiation include the latent membrane proteins, the EBERs, BNLF2a/BNLF2b, or simply the presence of viral DNA. Both LMP1 and LMP2A have been reported to affect epithelial cell differentiation and growth. Expression of LMP2A in foreskin keratinocytes was reported to activate phosphatidylinositol 3-kinase (PI3K), AKT, and β-catenin signaling and resulted in inhibition of differentiation by suspension in methylcellulose (27), and expression of LMP2A in HaCaT cells upregulated the expression of ΔNp63α, which is critical for the maintenance of basal keratinocytes (28, 29). LMP1 has been reported to affect the growth and morphology of epithelial cells (30), although LMP1 expression is very low in our cells. The EBERs are very strongly expressed in NOKs-Akata. While their function is poorly understood, they have been described to promote cellular growth and proliferation in GC (31) and NPC (32) cell lines. While BNLF2a has been characterized to have a role in immune evasion (33, 34), either gene may play a role in inhibiting keratinocyte differentiation.

By linking their replication cycles to epithelial cell differentiation, DNA tumor viruses, such as human papillomavirus (HPV) and EBV, ensure that virions are produced at epithelial surfaces, where they are best able to spread to other hosts. However, terminal differentiation presents challenges to these viruses in that the cells are exiting the cell cycle, undergoing extensive cross-linking via transglutamination of their cell cortex to cytokeratins, and ultimately becoming metabolically inert. Thus, it is not surprising that EBV has evolved strategies to attenuate differentiation to allow cellular processes to persist long enough for virions to be produced and shed. Previous work has shown that EBV induces an S-phase-like state when it begins lytic replication, allowing the replication of viral DNA (35), and that successful lytic activation depends on the activity of S-phase cyclin-dependent kinases (CDKs) (36). What is surprising is that EBV exerts these antidifferentiation effects prior to and independently of lytic cycle entry. This is consistent with prior results indicating that even transient EBV infection results in an impaired differentiation response to MC and calcium (10). We now extend these results and show that EBV exerts these effects in physiologic raft cultures and that this process does not require expression of the BARTs.

Cessation of the cell cycle is a key step in the terminal differentiation of keratinocytes (37) and can act as a failsafe to prevent uncontrolled proliferation of cells that harbor oncogenic mutations or overexpress oncogenes, such as the c-Myc gene (38). It has long been known that EBV-associated carcinomas typically exhibit defects in differentiation (39). These defects are likely a prerequisite for the maintenance of EBV in latency (40, 41), as cells capable of differentiation would be negatively selected due to lytic activation of the virus (8). Our results support the notion that EBV latent genes may present additional barriers to differentiation, which may contribute to the maintenance of latent EBV infection and continued cell cycle progression. Because undifferentiated NPC is almost always EBV positive, it is likely that EBV infection is an early and rate-limiting step in its development. A direct link between EBV infection and inhibition of differentiation may represent a critical step in allowing a cell to proliferate indefinitely. Understanding how EBV-infected keratinocytes avoid differentiation-associated exit from the cell cycle is crucial to understanding EBV’s role in carcinomagenesis.

## MATERIALS AND METHODS

### Cell lines and culture.

The hTERT immortalized normal oral keratinocyte (NOKs) cell line and derivative cell lines infected with the Akata strain of EBV (NOKs-Akata) have been previously described (4, 10). Additional uninfected control NOKs stably expressing enhanced GFP and G418 resistance were created by transfecting pEGFP-C1 linearized with ApaLI. The replication-defective AkataΔRZ genome was constructed in the Akata bacmid as previously described (9). All NOKs cell lines were maintained in an undifferentiated state by culturing them in keratinocyte serum-free medium (KSFM) supplemented with human epidermal growth factor and bovine pituitary extract (Life Technologies) and under selection with 50 μg/ml G418.

NOKs or NOKs-Akata were differentiated following trypsinization by resuspension in 1.6% methylcellulose (MC) dissolved in KSFM and placed in 50-ml conical tubes (Falcon) for 24 h at 37°C. Cells were collected after dilution in a 1:1 volume of phosphate-buffered saline (PBS) by centrifugation (1,500 rpm for 10 min) and washed with additional PBS. Cells were grown in organotypic raft cultures as described previously (42).

### Antibodies.

Primary antibodies included β-actin (C4; Santa Cruz Biotechnologies), involucrin (SY5; Sigma-Aldrich), Zta (BZ1; Santa Cruz Biotechnologies), Rta (produced by Pierce Biotechnology against peptide EDPDEETSQAVKALREMAD, as previously described [8]), K10 (PRB-159P; Covance), MCM7 clone 47DC141 (Thermo Fisher), and Itgβ4 (sc-9090; Santa Cruz Biotechnology).

### RNA-seq.

Cell pellets were harvested in RNA STAT-60, and RNA was isolated according to the manufacturer’s instructions. RNA isolated from 10^6^ cells seeded in a 60-mm culture dish served as an undifferentiated control. RNA from four independent biological replicates per condition was isolated. RNA integrity was assessed on an Agilent TapeStation 2200 using the RNA ScreenTape assay. Approximately 800 ng of total RNA was ribodepleted using the Ribo-Zero Gold reagent (Illumina). Sequencing libraries were prepared using the Illumina TruSeq stranded total RNA low-throughput (LT) kit according to the manufacturer’s instructions. Library size was determined using the Agilent TapeStation 2200 D1000 assay and quantified with the Quanta PerfeCTa NGS quantitative PCR (qPCR) quantification kit. Libraries were normalized to 4 nM, pooled, denatured, and diluted to approximately 1.8 pM. A PhiX library was spiked in as an internal control. A pool of 8 samples plus PhiX was single-end sequenced using an Illumina NextSeq 500 high-output cartridge (101-bp reads).

### Processing of the RNA-seq samples.

RSEM (version 1.3 [43]) and Bowtie (version 1.1.1 [44]) were used to quantify gene expression using the human genome assembly hg19. DESeq2 (version 1.2 [45]) was used to analyze differences in gene expression using read counts for each gene from RSEM, excluding genes with fewer than 20 aligned reads. *P* values were adjusted using the Benjamini-Hochberg method. For EBV-positive samples, using Burrows-Wheeler Aligner (BWA) (46), reads were aligned to the Akata genome (GenBank accession number KC207813.1), which was modified to reflect insertion of the G418-GFP sequence into either the *BXLF1* gene (47) or, in the case of the Akata bacmid, the BART locus. Alignments are summarized in Table S1 in the supplemental material. Wiggle tracks were generated from Sequence Alignment Map (SAM) alignment files and normalized for read length and millions of reads mapped (human plus EBV) to yield numbers of reads per kilobase of transcript per million mapped reads (RPKM). RNA-seq data from EBV-positive tumor samples from previously published studies (13, 14) were aligned to the type I EBV reference strain (GenBank accession number NC_007605.1) using BWA. Wiggle tracks were created using the RSEM rsem-bam2wig utility.

10.1128/mBio.01332-19.1 TABLE S1 Mapping statistics for RNA-seq data. For each RNA-seq sample, the total number of reads and the numbers of reads uniquely aligned to human (hg19) and Akata (for EBV-positive samples) are given. Download Table S1, XLS file, 0.02 MB. Copyright © 2019 Eichelberg et al. 2019 Eichelberg et al. This content is distributed under the terms of the Creative Commons Attribution 4.0 International license.

### GSEA.

Gene set enrichment analysis (GSEA) was performed using the Broad Institute’s GSEA implementation (48). Genes were sorted according to the signal-to-noise metric. All GSEA comparisons were run using the hallmark gene set (49) from the Molecular Signature Database (MSigDB) (50). One replicate was not included in GSEA (NOKs-Akata [no treatment] replicate 1) due to a low number of reads uniquely aligned to the human genome.

### Histology.

Formalin-fixed, paraffin-embedded raft sections were deparaffinized and then examined by hematoxylin and eosin and immunofluorescence (IF) staining. For IF staining, paraffin was removed in xylenes and slides were rehydrated in a series of ethanol washes followed by a PBS wash. Antigen retrieval was performed by microwaving the slides for 20 min in 10 mM sodium citrate (pH 6.0). After cooling, sections were blocked using 5% serum for 1 h and then incubated in primary antibody overnight at 4°C, followed by incubation in a 1:500 dilution of secondary antibody for 1 h and a series of PBS washes. Sections were counterstained with Hoechst 33342 dye. Secondary antibodies used were Alexa 488-conjugated donkey anti-mouse and Alexa 647-conjugated donkey anti-rabbit (Life Technologies). All images were taken with a Zeiss Axio Imager M2 microscope using the AxioVision software, version 4.8.2.

### Data availability.

All primary sequencing data have been submitted to the NCBI Sequence Read Archive (BioProject accession no. PRJNA555053).
