# Supplementary figures and images for: Epstein-Barr Virus Infection Promotes Epithelial Cell Growth by Attenuating Differentiation-Dependent Exit from the Cell Cycle
Source: mBio. 2019 Aug 20;10(4):e01332-19. doi: 10.1128/mBio.01332-19 (PMC6703421; doi:10.1128/mBio.01332-19)

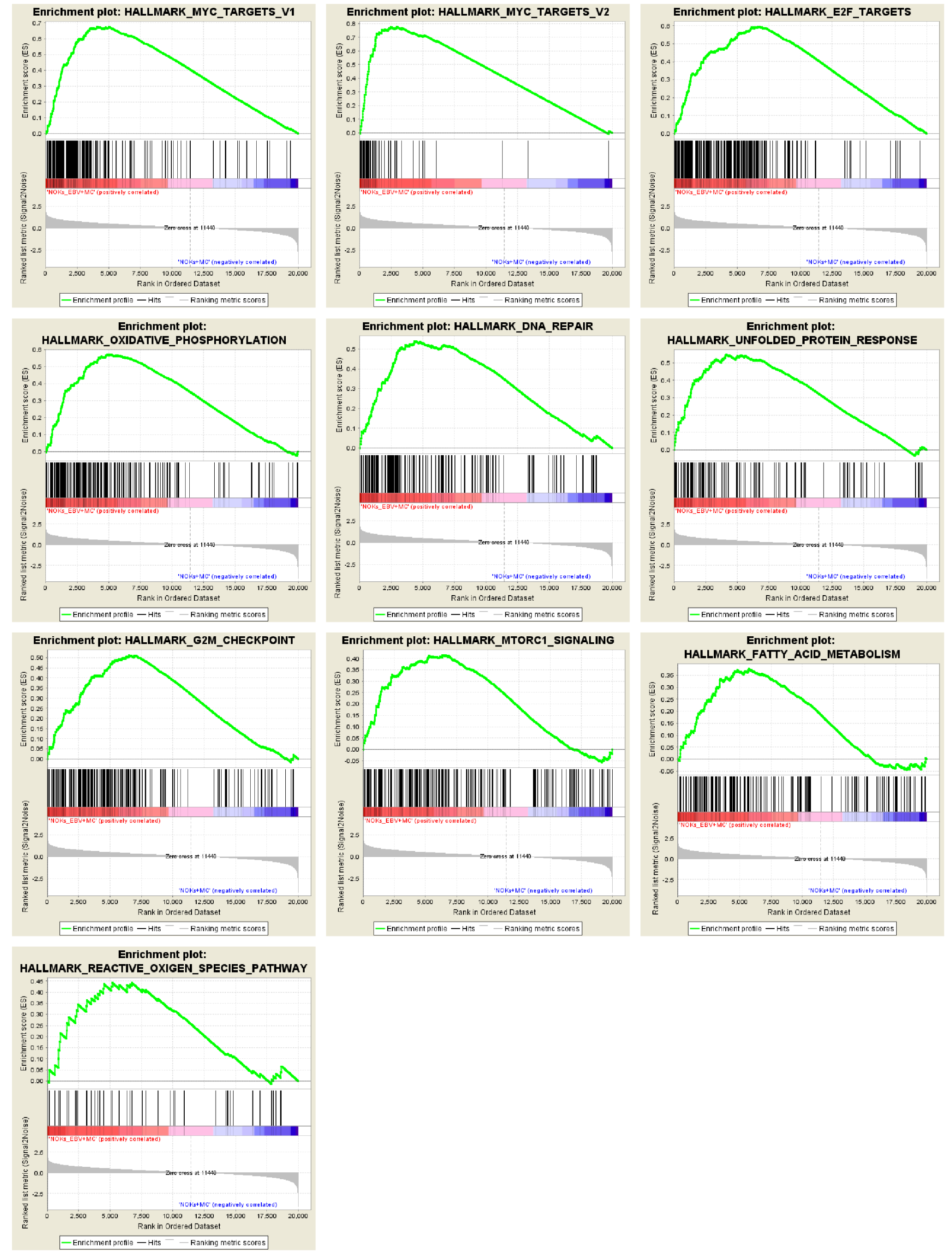

Supplement: FIG S3 [file mBio.01332-19-sf003.tif]

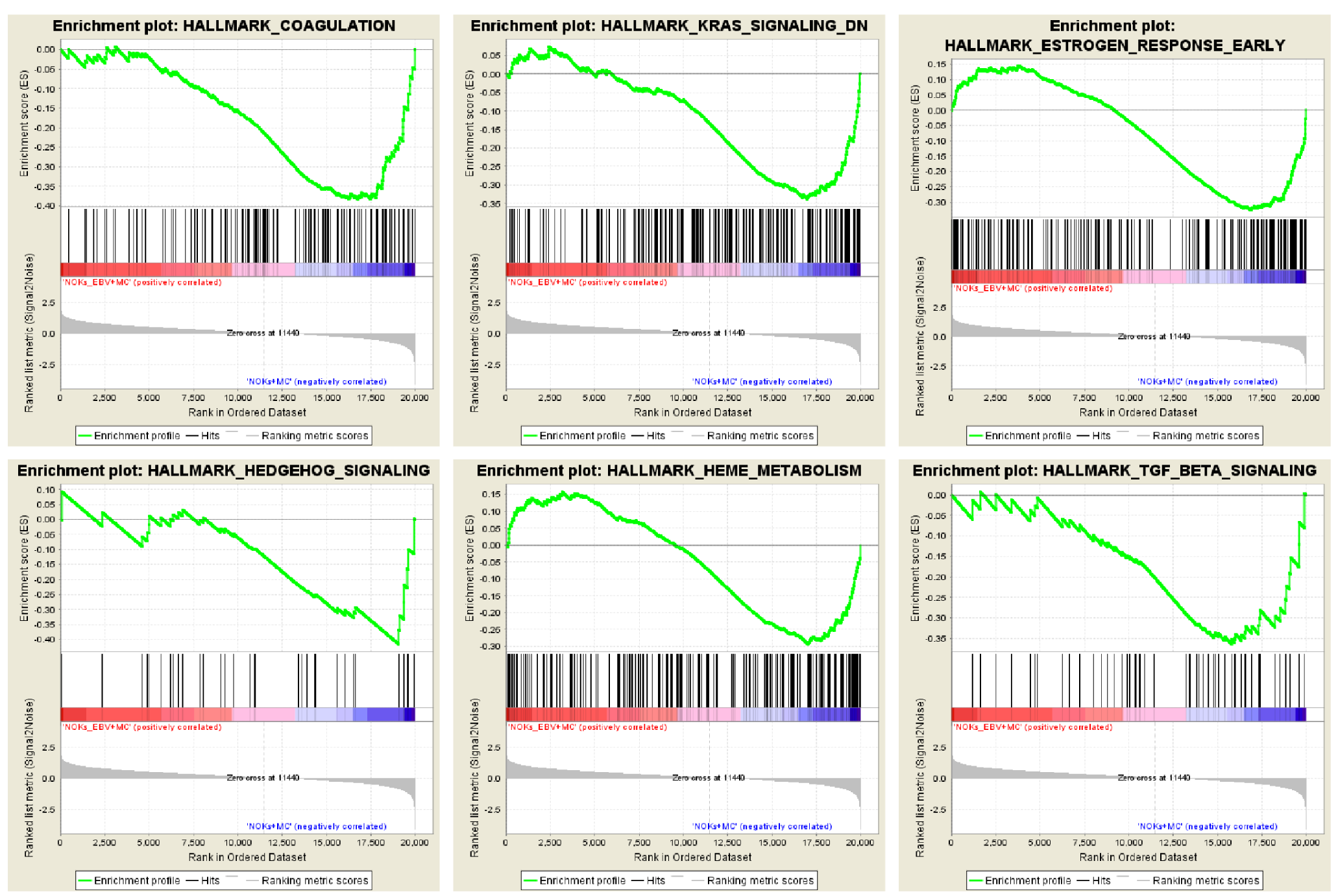

Supplement: FIG S4 [file mBio.01332-19-sf004.tif]
